# High-Efficiency Plasma Separator Based on Immunocapture and Filtration

**DOI:** 10.3390/mi11040352

**Published:** 2020-03-28

**Authors:** Xiaosong Su, Jianzhong Zhang, Dongxu Zhang, Yingbin Wang, Mengyuan Chen, Zhenyu Weng, Jin Wang, Juntian Zeng, Ya Zhang, Shiyin Zhang, Shengxiang Ge, Jun Zhang, Ningshao Xia

**Affiliations:** 1 State Key Laboratory of Molecular Vaccinology and Molecular Diagnostics, Xiamen University, Xiamen 361102,China; suxiaosong@xmu.edu.cn (X.S.); jzzhang@stu.xmu.edu.cn (J.Z.); zhangdongxu@xmu.edu.cn (D.Z.); ybwang@xmu.edu.cn (Y.W.); chenmengyuan@stu.xmu.edu.cn (M.C.); wengzhenyucup@163.com (Z.W.); wangjinxiada@163.com (J.W.); zengjuntian@126.com (J.Z.); zhang@stu.xmu.edu.cn (Y.Z.); sxge@xmu.edu.cn (S.G.); zhangj@xmu.edu.cn (J.Z.); nsxia@xmu.edu.cn (N.X.); 2 National Institute of Diagnostics and Vaccine Development in Infectious Diseases, Xiamen University, Xiamen 361102, China; 3 School of Public Health, Xiamen University, Xiamen 361102, China

**Keywords:** microfluidics application, plasma separation, point-of-care testing, sample preparation, separator, whole blood

## Abstract

The shortcomings of standard plasma-separation methods limit the point-of-care application of microfluidics in clinical facilities and at the patient’s bedside. To overcome the limitations of this inconvenient, laborious, and costly technique, a new plasma-separation technique and device were developed. This new separation method relies on immunological capture and filtration to exclude cells from plasma, and is convenient, easy to use, and cost-effective. Most of the RBCs can be captured and immobilized by antibody which coated in separation matrix, and residue cells can be totally removed from the sample by a commercially plasma purification membranes. A 400 µL anti-coagulated whole blood sample with 65% hematocrit (Hct) can be separated by the device in 5 min with only one pipette. Up to 97% of the plasma can be recovered from the raw blood sample with a separation efficiency at 100%. The recovery rate of small molecule compounds, proteins, and nucleic acid biomarkers is evaluated; there are no obvious differences from the centrifuge method. The results demonstrate that this method is an excellent replacement for traditional plasma preparation protocols.

## 1. Introduction

Microfluidics, the manipulation of reagents and samples on microchips, is a new strategy to improve existing methods and offer new approaches for analysis. Microfluidics techniques based point-of-care testing are now successfully used for chronic disease management and on-site analysis, such as glucose testing, uric acid testing, pregnancy testing, and infection disease detection [1,2]. Much effort has been made to extend the clinical applications of these techniques by developing integrable and easy-to-use sample preparation modules, e.g., for plasma preparation.

Plasma is an important clinical sample for chemistry and immunology testing, which still cannot be easily purified and recovered from whole blood samples using commercialized point-of-care-testing platforms. Currently, active and passive separation are two major types of microfluidics-based plasma preparation [3,4]. A dielectrophoresis-assisted plasma-separation device was developed by Liao et al. in 2013 [5]. As a typically active separation technique, it used two electrodes to enhance the aggregation of red blood cells (RBCs). By contrast, a passive separation technique based on the Zweifach–Fung effect has been developed to separate plasma from whole blood samples using specially designed microchannels [3,6,7,8,9,10,11]. The most common approach used in the past few years is passive filtration by pillar array [12,13,14,15,16,17,18,19,20,21] or implanted filtration membrane [22,23,24,25,26].

Although these efforts demonstrated the possibility of achieving microfluidics-based plasma purification, they are still limited by many deficiencies in technique. So far, reported designs are too complicated for massive production and system integration [6]. The low hematocrit requirement, normally around 10–45%, for sample input, is another common problem of the reported designs. Sample predilution is required to maintain the required hematocrit; testing sensitivity will be decreased by this sample pretreatment [7,9,27,28,29,30,31,32]. However, the aggregation of RBCs tends to block the sample flow pathways of filtration designs and results in low separation efficiency. Lee et al. [33] reported a filtration microdevice with a pulsatile module to agitate clogged cells and prevent pathway blocking, but its improvement effect in practice is far less than expected. A brief comparison of representative designs between different working principles was conducted and the results were listed in Table 1.

It can be observed that the huge number of RBCs is the main reason for most of the challenges in plasma separation. Samples with eliminated RBCs alleviate cell clogging, even without predilution. For this reason, removal of RBCs from a sample before filtering it is an ideal plasma-separation approach that is suitable for integration in microfluidics devices. In this study, a new blood separator has been designed and evaluated. This brand-new separator was based on immunological capture and filtration to exclude cells from plasma. Antibodies coated in a selected separation matrix captured RBCs while the sample flowed through, and the RBC-excluded plasma was filtrated by plasma separation membrane for residue cells removal. Owing to its huge internal surface area and surface–volume ratio, an acetate fiber pillar was used as the separation matrix which coated with anti-RBCs due to its high capturing capacity and separation capability. In addition, the matrix concentrates the sample flow trace, decreasing the complexity of the structure and the device fabrication, and will enable the device to be mass-produced, integrated, and commercialized. This immunocapture blood separator (IcBS) offered a convenient, easy to use, and cost-effective way for plasma sample preparation in field and medical laboratories.

## 2. Materials and Methods 

### 2.1. Materials and Reagents

The polymethyl methacrylate (PMMA) used in this research was purchased from Oudifu Inc., China. Double-sided sticky tape was purchased from SDK Inc. China. Xtreme-Xtra smoking filter tips were purchased from Smoking Planet SL, Spain, and used as the separation matrix in this research. Anti-RBC was obtained from Wantai BioPharm Inc, China. and preserved in −20 °C. Pall™ Vivid™ GR plasma-separation membrane, purchased from GE, US, was used as the purification membranes.

A quantitative assay kit for ferritin was purchased from Wantai BioPharm Inc., and a glucose point-of-care testing kit was purchased from Sannuo, China. A hepatitis B virus (HBV) viral loading testing kit was purchased from Sansure Biotech, China.

### 2.2. Samples

Blood samples were collected from the researcher and the collaborators. Blood sample tubes pretreated with heparin (BD, USA) were used to prevent coagulation. All these samples were collected and tested while fresh; no preserved samples were used in this study. Independent ethics committee approval was obtained from the Ethics Committee of the National Institute of Diagnostics and Vaccine Development in Infectious Diseases, China.

By contrast, HBV-positive blood samples, which were used in nucleic acid recovery evaluation, were collected from Xiamen Traditional Medicine Hospital. Permission was obtained from the hospital’s ethics committee, and written informed consent was provided by all participants.

### 2.3. Methods

#### 2.3.1. Production of Immunocapture Matrix

An acetate fiber pillar of 6.0 mm diameter was cut to 11 mm in length before antibody coating. The matrix was then soaked in anti-RBC solution with a concentration of 0.4 mg/mL. The matrix was removed from the solution after 2 h incubation and quickly transported to −80 °C for freezing, followed by overnight lyophilization. Antibody-coated separation matrix was then preserved at room temperature for further application.

#### 2.3.2. Device Design and Fabrication

The microdevice was designed and fabricated from PMMA and double-sided sticky tape using standard fabrication techniques. Briefly, the components of the microdevice were designed using AutoCAD 2014 software (Autodesk, USA). Then, PMMA coated with double-sided sticky tape was cut using a Spirit SI-30Ti laser cutter according to the AutoCAD blueprint. The chip was sealed with the tape and the bonding was reinforced by quick heat pressing at 500 N and 60 °C for 5 min. After the whole microdevice was assembled, the pillar matrix was inserted into the primary separation chamber, making sure that the matrix connector was inserted into the matrix.

#### 2.3.3. Sample Preparation for the Control Group

The plasma sample was separated from the anti-coagulated whole blood sample by 3000 rpm centrifugation for 10 min. This procedure has served as the gold standard in clinical laboratories for decades. Plasma sample was recollected and stored at 4 °C for further evaluation.

#### 2.3.4. Cell counting

A Mindray BC-1800 automatic blood cell analyzer was used to count cells, to evaluate the residue cells in the plasma sample.

#### 2.3.5. Glucose Analysis

Plasma glucose analysis was performed using a Sannuo GA-3 glucose point-of-care analyzer. Briefly, a test strip was inserted into the analyzer, and a drop of plasma sample was applied at the end of the strip. The analyzer determines and displays the result automatically.

#### 2.3.6. Ferritin Analysis

Plasma ferritin was analyzed using a quantitative assay kit, according to the manufacturer’s instructions (Wantai BioPharm Inc.). Briefly, 20 µL of plasma sample was mixed and incubated at 37 °C for 15 min with 50 µL of antibody-coated magnet particles, followed by three washes. Then 50 µL of acridinium-ester-labeled antibody was applied and incubated for 10 min. After four washes, prestimulant and stimulant reagents were added and a light signal was detected within 3 s. A standard curve for the ferritin assay was constructed by testing standard controls in a two-fold dilution.

#### 2.3.7. DNA Analysis

A one-step commercial HBV viral load assay kit (Sansure Biotech, China) was used in this evaluation, according to the manufacturer’s instructions. Briefly, 5 µL of plasma sample was mixed with 5 µL of nucleic acid release reagent. After 10 min incubation at room temperature, 40 µL of amplification reagent, containing primers, probes, inner control, and enzymes, was added to and mixed with the sample. The system was immediately analyzed using real-time polymerase chain reaction (PCR) by the Bio-Rad CFX86 Fluorescence Thermo Cycler, with the following conditions: initial denaturation at 94 °C for 5 min, followed by 45 cycles at 94 °C for 15 s and 55 °C for 30 s. A standard curve for the HBV virus assay was created by testing standard controls included with the assay kit.

#### 2.3.8. Statistics

Experimental data were analyzed and plotted using GraphPad Prism version 7.00.

## 3. Results

### 3.1. Microdevice Functioning Design

**Figure 1:**
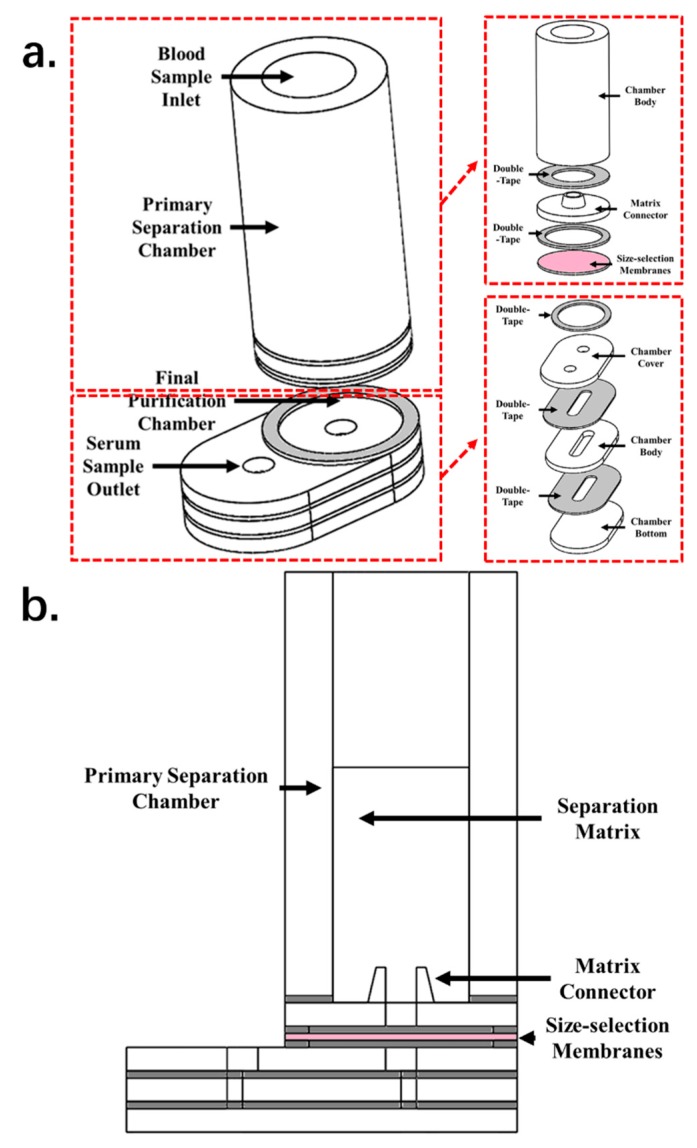
Microdevice: (**a**) device fabrication; (**b**) sectional view.

As shown in Figure 1, the separation microdevice is composed of two major chambers—a primary separation chamber and a final purification chamber. The primary separation chamber holds the separation matrix and serves as a container for the matrix and blood samples. The final purification chamber is connected to the primary separation chamber and holds the filtration membranes. The purified plasma sample can be collected from a fluid channel directly connected with the final purification chamber. The primary separation chamber is cup-shaped and supports the acetate fiber pillar matrix. The inner diameter of this section is 6 mm, and the height is 18 mm. This size is sufficient to hold an 11 mm high separation matrix and about 400 µL of whole blood. The core functioning section of the purification chamber consists of the filtration membranes. A membrane of 8 mm diameter is placed in the center of the chamber. To offer space for the preseparated sample to contact the filtration membranes, gaps of 0.3 mm in height were left on each side of the membranes, to allow sample contact with the membranes and enhance the separation efficiency.

The primary separation chamber and the purification chamber are connected by a short sample-conducting channel with a 2 mm high needle structure that penetrates the separation matrix and improves sample volume recovery by focusing the vacuum pressure. Another conducting channel, 14 × 1 × 1 mm was used to guide the purified plasma sample to the collecting port. Thus, with the purification chamber, the calculated dead volume of the device is about 22.48 µL.

### 3.2. Device Operation

**Figure 2:**
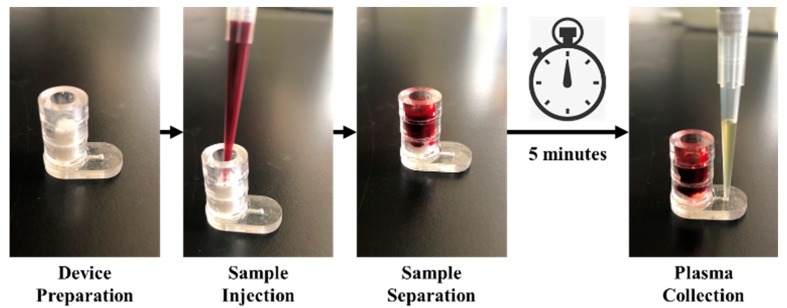
Device operation process.

As shown in Figure 2, 400 µL of whole blood sample was injected into the microdevice; then the sample was let flow through the separation matrix under gravity for 5 min to allow cell capture. A 200 µL pipette was used to collect purified plasma sample directly from the outlet. Collected plasma samples were preserved in 1.5 mL tubes at 4 °C for further evaluation.

### 3.3. Evaluation of Separation Matrix Function

**Figure 3:**
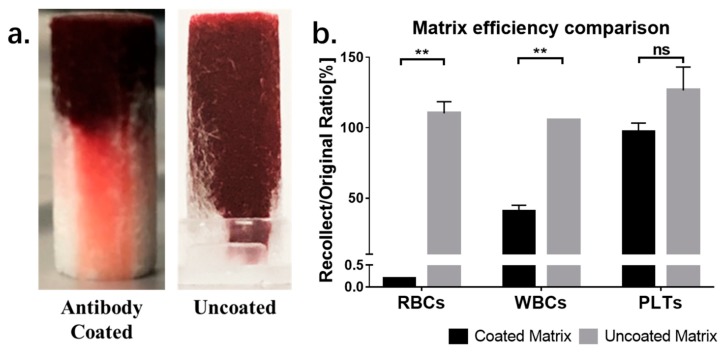
(**a**) Anti-RBC coated separation matrix can separate plasma from whole blood sample by immobilizing cells in the matrix. (**b**) Matrix separation efficiency comparison between coated and uncoated acetate fiber pillar. PLT, platelet; RBC, red blood cell; WBC, white blood cell. ** means *p* < 0.01, ns means *p* > 0.05.

A test was conducted to evaluate the separation efficiency of the acetate fiber matrix. First, we compared the separation efficiency between antibody-coated and uncoated matrix. As shown in Figure 3, no separation can be observed in uncoated matrix. The whole blood sample flowed all the way through the matrix, without separation. By contrast, using the antibody-coated matrix, RBCs can be captured in the top half of the separation matrix and the plasma sample can be directly collected by pipetting. A cell count was also conducted to evaluate the separation efficiency. The percentage was calculated by divided cells counted from the whole blood sample by cells counted from samples collected from coated or uncoated matrix. As shown in Figure 3b, compared with whole blood sample, samples from uncoated matrix failed to exclude any cells from whole blood sample, while the numbers of residual RBCs and white blood cells (WBCs) were significantly decreased after separation by the coated matrix. It also can be observed that samples recollected from the uncoated matrix showed a higher percentage of cells than the original blood samples. This may be due to the increased cell concentration of recollected samples, caused by the plasma absorption effect in the matrix.

**Figure 4:**
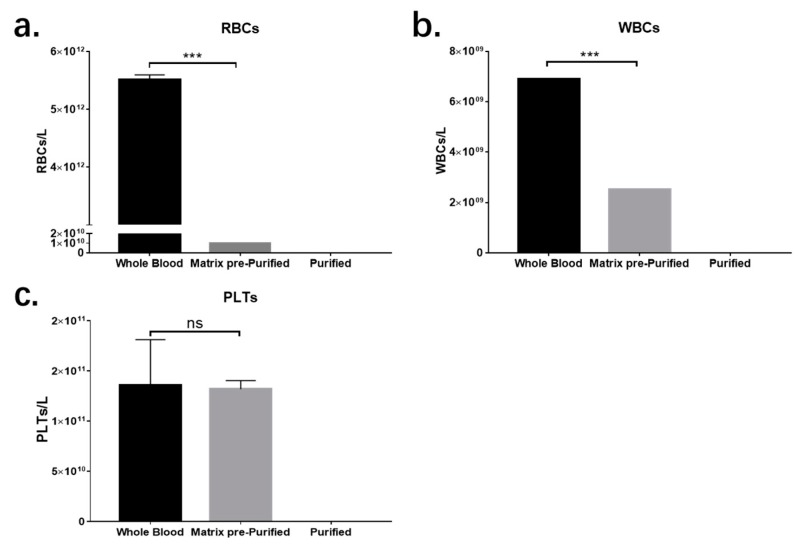
(**a**) Red blood cell (RBC) count, (**b**) white blood cell (WBC) count, and (**c**) platelet (PLT) count from whole blood sample, matrix prepurified sample, and purified sample. *** means *p* < 0.005, ns means *p* > 0.05.

Using the antibody-coated matrix, the exclusion efficiencies of RBCs, WBCs, and platelets were 99.8%, 60%, and 0%, respectively. The low exclusion rates of WBCs and platelets may due to the channel size of matrix, which is large enough for cells to travel through and the RBC-specific antibody, which cannot immobilize other cells. Although residue cells can still be detected in separated plasma samples collected from the matrix, all of these will be removed after filtration purification. The results are shown in Figure 4.

The volume recovery capabilities of matrixes of different lengths were also assessed to determine the most suitable dimensions of the separation matrix; separation matrix lengths of 12, 10, 7.5, and 5 mm were chosen. The loading volumes for matrixes of different lengths were calculated as the total volume of the pillar (ttV). Samples with 20%, 40%, 60%, 80%, and 100% of ttV were tested to calculate the dead volume of the matrix and determine the best loading volume of the sample. Plasma samples failed to recollect from the plasma group for all the trials at 20% ttV, and from the whole blood group for all trials at 20% and 40% ttV. The average dead volume among the different matrixes for plasma and whole blood were 4.33 ± 0.10 µL/mm and 0.58 ± 0.12 µL/mm, respectively.

It can be observed that the dead volume for the plasma sample in each matrix length group is significantly larger than that for the whole blood sample. When the plasma sample was loaded into the matrix, it quickly diffused inside the matrix. After plasma recollection, most, but not all of the sample can be recovered. Therefore, the dead volume calculated using the plasma sample is the “true dead volume” of the matrix; it reflects the amount of sample that cannot be recovered from the matrix.

However, referring to the whole blood sample, the situation is totally different. During the process of whole blood diffusion in the matrix, blood cells and plasma will be separated, owing to the antibody capture. The blood cells fall behind and occupy most of the dead volume. The more packed the blood cells are, the smaller the dead volume for plasma.

Another problem is the total loading volume of the sample. According to the result, the total loading volume should be greater than 60% ttV to ensure that there is enough sample to fill the matrix. The upper limit loading volume should be considered according to the patient’s hematocrit and the volume of packed cells, but is normally less than 150% of the ttV. The designing parameters such as diameter and height of the matrix can be changed based on the requirement of output volume.

### 3.4. Evaluation of Recovery Parameters

**Figure 5:**
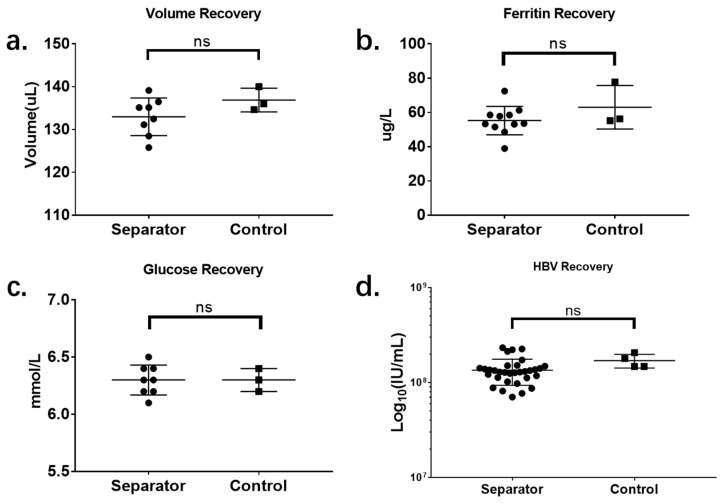
Evaluation of the separation microdevice, compared with centrifuge control: (**a**) volume recovery; (**b**) ferritin recovery; (**c**) glucose recovery; (**d**) hepatitis B virus (HBV) recovery. ns means *p* > 0.05.

As shown in Figure 5, four parameters were analyzed to evaluate the performance of the microdevice. The first parameter, volume recovery, refers to the percentage of plasma that can be recovered from the microdevice, compared with the centrifuge control; this directly represents the plasma-separation efficiency of this device. Using the centrifuge control, 136.9 ± 1.60 µL of plasma was collected from 400 µL sample, while 131.84 ± 3.40 µL of plasma was collected using the microdevice. The plasma volume recovery rate is 97.14% ± 1.134%.

The second evaluation parameter is ferritin recovery, which represents the recovery efficiency of the protein biomarker in plasma samples. In the control plasma, 63.03 ± 7.34 µg/L of ferritin was detected, while the result for the test group was 55.23 ± 2.52 µg/L. The ferritin recovery was 87.12% ± 1.83%.

For the evaluation of the recovery efficiency for non-protein biomarkers, the plasma glucose level in the plasma sample was tested. In the control, 6.3 ± 0.06 mmol/L of plasma glucose was detected, while the result for the test group was 6.3 ± 0.05 mmol/L. The recovery rate for plasma glucose, which represents the most commonly tested clinical chemistry biomarker, is 100% ± 0.73%.

Quantitative testing of HBV was conducted to determine whether the separation device would affect viral load testing. The resolution is normally 1 Ct in traditional quantitative PCR, which can lead to a variation among 0.5–2× in quantitative results. This effect will terribly affect the recovery efficiency evaluation. The results were presented by calculating log10(IU/mL), which can directly show the order of magnitude of the HBV viral load without “resolution effects” caused by the PCR machine itself. The result for the centrifuge control is 8.23 ± 0.03, while that for the test group is 8.11 ± 0.02.

### 3.5. Evaluation of Recovery Variation

**Figure 6:**
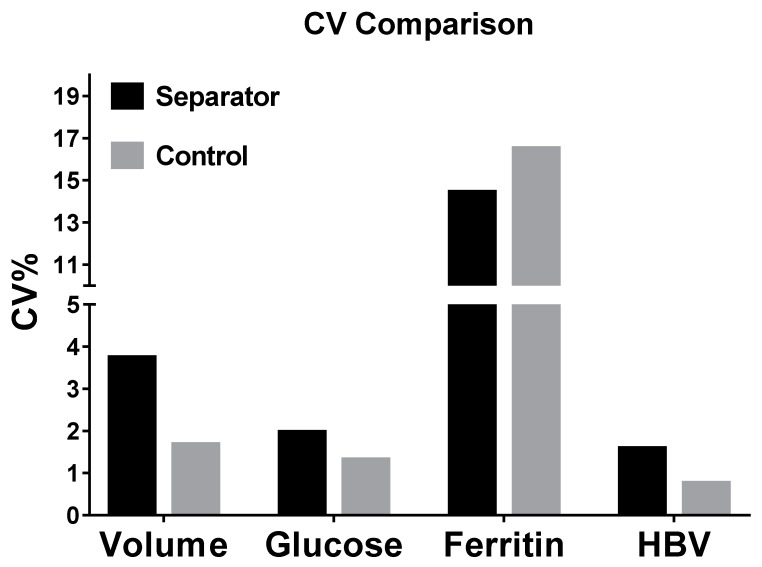
Percentage coefficient of variation (CV%) of all the four separation parameters. HBV, hepatitis B virus.

For variation evaluation, the percentage coefficient of variation (CV%) for all four separation parameters is presented in Figure 6. The CV% values of volume recovery, glucose recovery, and HBV recovery from IcBs were 3.72, 1.94, and 1.56, respectively, while those from the control were 1.66, 1.29, and 0.73, respectively. These differences may be caused by the differences between microdevices. In this study, the IcBs were manufactured manually using a standard PMMA-double-sided sticky tape fabrication technique. This technique is easy and economical, but will cause differences between devices because of manufacturing variation. Although the variation among the results from the IcBs is slightly higher than for those from centrifuge controls, it is still less than 5 and can be considered acceptable according to the requirements of the Clinical Laboratory Improvement Amendments. Conversely, the CV% values of ferritin testing were 14.40% and 16.47% for IcBs and control, respectively. Those are much higher than for the other three parameters. This may be due to defects in the methodology. Differently from glucose testing and HBV testing we used in this research, the ferritin assay we used was performed manually. The random error and operation bias those caused by manual operation during testing will lead to an increased relative error. This problem can be solved after the application of an automatic immunoanalyzer.

## 4. Discussion

The complexity of fabrication, integration, and application are major challenges faced in microfluidics-based plasma preparation approaches reported in the literature. These problems limit the industrial production, commercialization, and clinical application of microfluidics-based plasma separators. To overcome the technical limitations of microfluidics-based plasma separation, a new plasma separator based on a combination of immunological capture and size-selection filtration was proposed and evaluated. Unlike other posted researches, IcBS shows great advantages in both performance characteristics and application characteristics.

**Figure 7:**
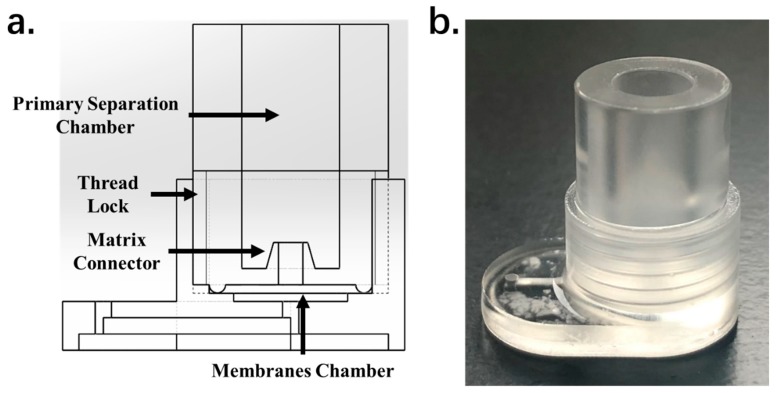
(**a**) The blue print and (**b**) the product of the IcBS produced by machining.

The IcBS successfully decreased the difficulty and cost of device production. Lithography and polydimethylsiloxane (PDMS) molding are the most commonly used fabrication techniques for microfluidic chip production. However, these methods are very complicated, environment restricted, and expensive. The quoted price for a single PDMS chip with a simple T-junction reaction channel is about $30–60. Compared with traditional plastic molding techniques, PDMS molding is not cost-effective. Although traditional molding techniques are the most convenient and cost-effective methods for mass-production, structures at the micrometer scale are beyond the production capabilities of plastic molding techniques. The IcBS was designed to be fabricated and operated in the millimeter scale, which can be easily mass-produced using traditional molding techniques. This improvement can significantly increase the productivity and decrease the cost. The IcBS is fabricated from PMMA and double-sided sticky tape, which, in this study, only costed about $2–3 per test. This cost can be greatly decreased to about $0.1 per test if the device is mass-produced using traditional molding techniques. We redesigned and assembled a new batch of IcBS and performed preliminary tests using machined parts in accordance with the requirements of mold production. Only two isolated parts which can be easily produced are required for the fabrication of IcBS through molding or machining. The performance characteristics show no significant differences between the machined one and the one which fabricated by PMMA-double tap technique. The blue print and the product of the IcBS produced by machining are shown in Figure 7. 

Another great advantage of the IcBS lies in the optimization by sample type. A whole blood sample with any hematocrit can be directly separated using the IcBS. In most blood plasma separator research, the sample Hct is usually below 45% [3,4]. But it can be noticed that the sample’s Hct in our work was around 65%, which is even slightly higher than the normal range. This means that sample pretreatment, such as predilution or reagent treatment, is not required. This improvement not only decreases the complexity of device application, but also increases the sensitivity and accuracy of the test. Handling errors and operation bias during sample pretreatment, such as barcode reading errors or pipetting bias, can also be avoided. This improvement also decreased the operation complexity. The only operations required of clinical technicians are sample injection and plasma sample recollection. This greatly enhances the acceptance of the microfluidics-based plasma purification technique and expands its clinical application. According to an informal survey of four clinical laboratory supervisors and twelve clinical laboratory technicians, the IcBS is more acceptable, owing to its convenient operation protocol and satisfactory purification efficiency.

Although a pipette was used in our laboratory, it is not the only choice for IcBS operation. Any equipment which can generate vacuum pressure can be used as the replacement in for the IcBS operation, such as a disposable dropper, a syringe, or elastic pumps. IcBS can also be connected to a lateral flow strip directly. In this case, plasma recollection will be accomplished by the capillary force of the strip automatically.

Its potential for universal application is another great advantage of the IcBS. Unlike other reported designs, the working principle of the IcBS is a combination of immunology and physics, and is based on a simple “flow through and recollection” process. The driving force for IcBs is a constant vacuum pressure, which has been widely used to drive fluid flow in microfluidics devices. The simple working principle and structure of the IcBS can be easily integrated with most existing microfluidics chips and serves as an effective sample preparation module without specially designed channels or connectors. It has also been proven that the separation capacity is directly related to the inner volume of the matrix; the plasma volume can be adjusted to fulfill different researchers’ requirements by simply changing the dimensions of the matrix. The universal application potential is well supported by these characteristics.

As well as microfluidics system integration, the IcBS can be directly used as a sample preparation device to offer high-quality plasma samples for any kind of clinical analysis. It can be used in facilities with limited medical resources where centrifuges are not affordable, such as community medical services, personal clinics, or village doctors’ practices. Conversely, the IcBS can also be used for special medical service fields, such as battlefield medical services, microorganism identification during infectious disease separation, and biomedical experiments in space stations. Owing to its easy preservation and application characteristics, it is an ideal complement for the traditional plasma purification method.

## 5. Conclusions

In this study, we have successfully developed a modular universal plasma-separation microdevice based on immunocapture and size filtering. This device was used in this study to separate 400 µL of whole blood sample to produce more than 100 µL of plasma without residue cells, but the separation capacity can be adjusted to fulfill different testing requirements. The final plasma output volume depends on the volume of whole blood sample input and its hematocrit. This microdevice is also very cost-effective and easy to use, costing as little as $2.00–3.00 per test in this study and requiring only three simple steps to separate raw blood samples in 5 min.

The IcBS offers a brand-new methodological option for plasma sample preparation. It is an ideal supplement for traditional blood sample processing techniques in resource-limited areas and medical facilities with limited budgets. It can also be integrated with other testing techniques or microfluidics-based assays as a sample preparation module and offers high-quality plasma samples.

Moreover, the IcBS can easily be mass-produced using traditional molding techniques. This improvement will reduce the cost of this microdevice to $0.1 per test, which will greatly benefit clinical laboratories’ operations and patient diagnostics in resource-limited areas after commercialization.

## Figures and Tables

**Table 1:** Performance characteristics comparison of representative designs for different working principles.

Performance Characteristics	Zweifach–Fung Effect [6]	T-Junction [9]	Filtration [22]	Enhanced Filtration [33]
Hct (%)	~45	~45	~45	15
Separation Efficiency (%)	78	42–99	Not applicable	100
Plasma Yield (%)	~7	1.8	70	90
